# Investigating medicinal resource combinations in the Bornean orangutan diet

**DOI:** 10.1038/s41598-026-52614-4

**Published:** 2026-05-13

**Authors:** G. Allen, E. Freymann, J. d’Oliveira Coelho, H. Shagara, I. Shinyo, A. Panda, A. Jaya, K. J. Hockings, H. C. Morrogh-Bernard

**Affiliations:** 1 Centre for Ecology and Conservation, Faculty of Environment, Science and Economy, University of Exeter, Penryn Campus, Penryn, Cornwall, TR10 9FE UK; 2 Institute at Brown for Environment and Society, Brown University, Rhode Island, USA; 3 Ethnopharmacology and Zoopharmacognosy Junior Research Group, Bernhard Nocht Institute for Tropical Medicine, Hamburg, Germany; 4 Primate Adaptations, Landscapes and Evolutionary Origins (PALEO), CIBIO - Universidade do Porto, Vairão, Portugal; 5 Science Department, Gorongosa National Park, Sofala, Mozambique; 6 Interdisciplinary Center for Archaeology and Evolution of Human Behavior (ICArEHB), Universidade do Algarve, 8005-139 Faro, Portugal; 7 Borneo Nature Foundation (BNF) Indonesia, Yayasan Borneo Nature Indonesia, Jalan Bukit Raya No. 17, Palangka Raya, Central Kalimantan 73112 Indonesia; 8 University of Palangka Raya, Jl. Yos Sudarso, Palangka, Kec. Jekan Raya, Kota Palangka Raya, Kalimantan Tengah 74874 Indonesia; 9 Tremough Innovation Centre, Borneo Nature Foundation, Penryn, Cornwall, TR10 9TA UK

**Keywords:** *Pongo pygmaeus*, Zoopharmacognosy, Self-medication, Food combinations, Feeding ecology, Ecology, Ecology, Plant sciences, Zoology

## Abstract

Self-medication occurs across diverse taxa, but identifying novel behaviours and medicinal resources remains challenging due to the need for long-term observation, intensive health monitoring, and costly pharmacological analyses. Recent advances in analytical methods have enabled the development of the self-medicative resource combination hypothesis (SMRCH), which proposes that non-human animals may deliberately combine therapeutic resources when ill. This framework has previously been applied to identify non-random dietary combinations in wild chimpanzees. Here, we extend this approach to another great ape by examining dietary combinations in wild Bornean orangutans (*Pongo pygmaeus*), a species with emerging evidence of medicative feeding behaviour, thereby enabling broader comparative insight into medicative feeding across great apes. Using long-term feeding data from a Sebangau peat-swamp forest Central Kalimantan, we analysed patterns of plant use and evaluated their potential medicinal relevance. Our analyses revealed non-random dietary combinations involving plant species with documented ethnomedicinal and pharmacological properties. These findings are consistent with the SMRCH, supporting its utility as a framework for identifying non-random dietary associations that may represent candidate self-medicative behaviours in orangutans and other great apes. This research highlights the importance of preserving Indigenous knowledge for biodiversity conservation and global health research.

**Supplementary Information:**

The online version contains supplementary material available at 10.1038/s41598-026-52614-4.

## Introduction

Zoopharmacognosy refers to the study of non-human medicinal behaviours, in which non-human animals therapeutically treat illness or injury in themselves or others^1,2^. Investigations into non-human self-medication have established the presence of diverse medicinal behaviours in a range of taxa including, but not limited to, birds, insects, reptiles and mammals^1,2^. Non-human medicinal behaviours can involve several modes^2^, including behavioural avoidance^3–5^, fumigation^6^, topical anointment for an external condition^7–9^, and the ingestion of resources with chemical or mechanical therapeutic properties^3,9–11^.

Throughout the animal kingdom, research into great apes, particularly chimpanzees (*Pan troglodytes),* has provided the most valuable insights thus far into non-human self-medication behaviours^1,12,13^. For example, several ingestion-based behaviours have been identified in chimpanzees, including leaf swallowing^14^ and bitter-pith chewing of the plant *Vernonia amygdalina*^15,16^*.* Both behaviours have been shown to reduce internal parasite infections^17^. Several lines of evidence led to the establishment of these behaviours as forms of therapeutic self-medication. These included the observation of chimpanzees with high parasite loads engaging in feeding on these plants, the lack of obvious nutritional value these behaviours offered consumers, and the seasonality of these behaviours corresponding with periods in which parasite infections are known to increase^15,16^.

Following the discovery of self-medication in chimpanzees, similar ingestion-based anthelminthic behaviours were also observed in other apes, including bonobos (*Pan paniscus*)^9–18^, gibbons (*Hylobatidae* spp.)^11,19^, and gorillas (*Gorilla beringei*)^20^. While orangutans (*Pongo spp*.) have been found to occasionally consume leaves with known medicinal properties, these behaviours have not yet been linked to the presence of any particular illness in orangutan consumers^21,22^. Orangutans are the only great ape who have not been observed leaf swallowing^19^.

However, despite the current absence of ingestion-based self-medication behaviours in orangutans, other behaviours have been suggested, providing evidence that they have the capacity for modes of self-medication. For example, Largo^23^ observed orangutans at Tuanan, Central Kalimantan, Indonesia, preferentially nesting in tree species with insect-repellent properties during periods of elevated mosquito density. In Sebangau, Central Kalimantan, Indonesia, adult female orangutans were observed mixing saliva with chewed *Dracaena cantleyi* leaves and applying the lather to specific parts of their bodies^24^. Subsequent analysis confirmed the plant’s anti-inflammatory properties, suggesting this behaviour may chemically relieve muscle or joint discomfort, possibly caused by the strain of carrying offspring^6^. Local indigenous communities also use leaves of this species as a poultice for pain relief. Kanamori et al.^26^ documented a severely injured juvenile Bornean orangutan in Danum Valley that spent approximately 1 h and 40 min selectively consuming pith of Zingiberaceae plant species, suggesting a potential self-medicative response to its traumatic injury, although the individual ultimately succumbed to the wound. Most recently, Laumer et al.^7^ reported the first documented case of a wild Sumatran orangutan using leaves from a medicinal liana, *Fibraurea tinctoria*, to actively treat a facial wound. The leaves of this tree are also known to have traditional medicinal uses^7,25^.

Despite recent progress in animal self-medication research, several limitations have historically constrained systematic long-term study of this topic. Given that therapeutic medicinal behaviours are often rare, studying these events has necessitated long-term studies with opportunistic observations. Recently, however, new methods have been proposed which offer alternative ways to study non-human medicinal diets using pre-existing long-term feeding data^27^.

In 2024, Freymann et al.^27^ introduced novel methods to explore potential medicinal resource combinations in nonhuman primate (hereafter, primate) diets, introducing a new hypothesis called the Self-Medicative Resource Combinational Hypothesis (SMRCH). The SMRCH represents a holistic advancement in how we think about health management in primates. This hypothesis posits that primates, in response to illness, may not rely on a singular medicinal resource for recovery, but rather combinations of resources which optimise therapeutic outcomes. By integrating multiple resources, individuals may potentially exploit synergistic or complementary interactions that enhance the overall efficacy of their self-medication strategies. This approach assumes a sophisticated health management strategy on behalf of the individual, as well as a nuanced understanding of how various substances collectively improve therapeutic effectiveness. After examining the feeding ecology of the Sonso chimpanzee group in the Budongo Forest of Western Uganda, Freymann et al.^27^ were able to identify several putatively medicinal resource combinations which occurred within wild chimpanzee diets. They were also able to show that many of these medicinal resource combinations occurred in the diet non-randomly, suggesting the presence of intentional selection. While proof of these medicinal combinations requires targeted chemical analyses to identify bioactive compounds and additional behavioural data, the feeding data can be analysed to detect non-random resource combinations, identifying species for further investigation.

The SMRCH further challenges models that focus on single-resource use, drawing parallels between primate behaviour and human healthcare practices^27^. Much like systems of modern Western medicine, research in Zoopharmacognosy has thus far emphasised the use of single medicinal resources^13,27^. While this approach has been effective in identifying novel cases of self-medication, the focus on singular medicinal resources may cause additional items in the diets of sick individuals to be overlooked. Investigating the SMRCH in Bornean orangutans addresses a gap in great ape self-medication research, as medicinal resource combinations have not previously been systematically examined in this species. However, anecdotes suggest that medicinal combinations may appear in their diets^28^.

Here, we use a 20-year database on orangutan feeding ecology collected through focal observations and apply Multiple Distinctive Collocation Analysis (MDCA)^29^ and APRIORI^30^ analysis to determine whether specific resource combinations, specifically those involving at least one resource with known or suspected medicinal properties, occur more frequently than would be expected by chance^27^. By leveraging the extensive temporal scope of our dataset, this study aims to provide novel insights into Bornean orangutan self-medication behaviours.

## Methods

### Study site and subjects

**Fig. 1:**
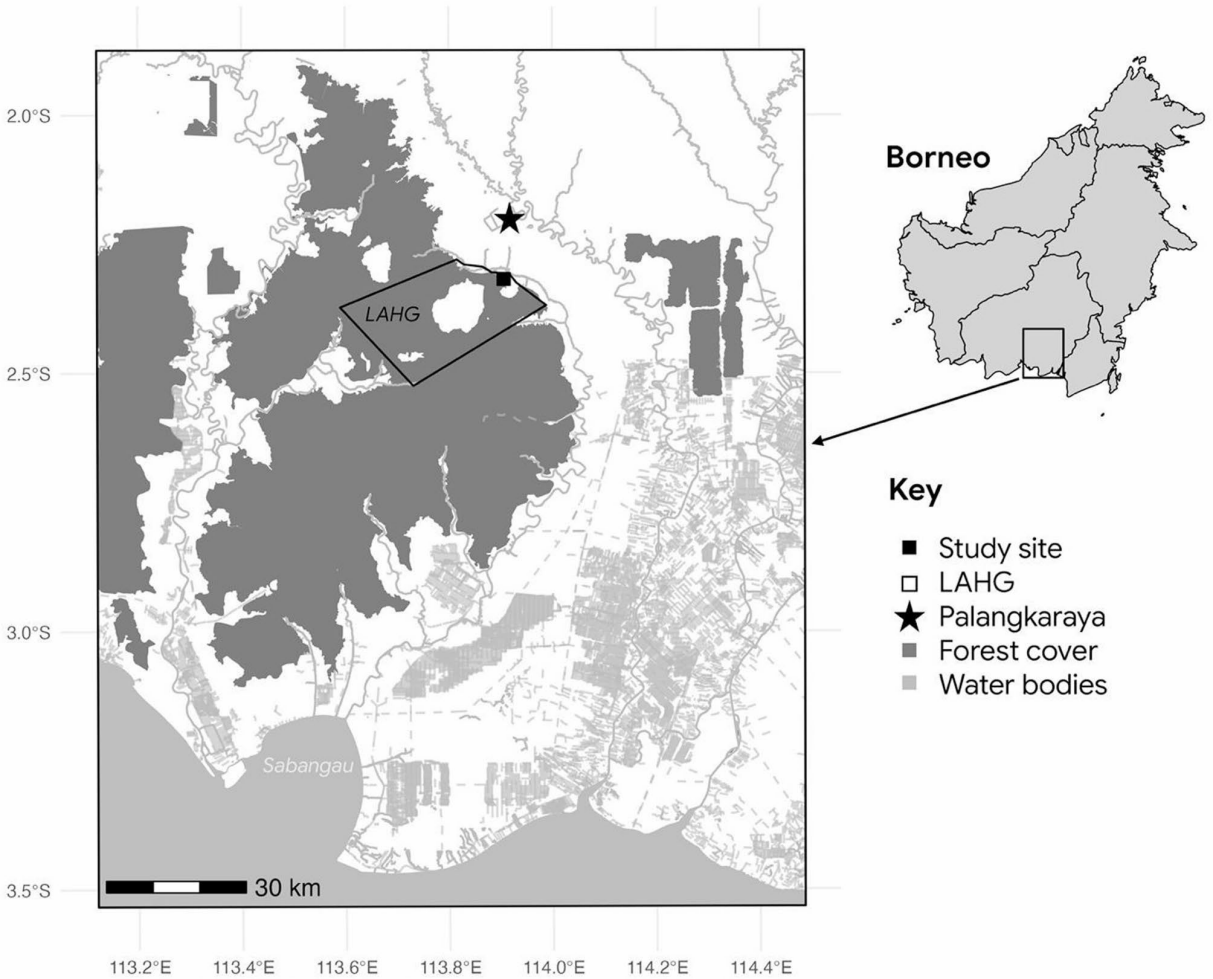
Location of the 3 km × 3 km study site (black box) within the Laboratorium Alam Hutan Gambut: Natural Laboratory (LAHG) for the Study of Peat Swamp Forest. This is a 500 km^2^ area in the Sabangau Forest, and was designated for the purpose of scientific research in 1997. Map created by co-author J. d’Oliveira Coelho using R (v4.5.2). It was generated using publicly available shapefiles; data and code are available at: https://github.com/Delvis/Borneo_maps.

Research was conducted within the Kawasan Khusus, a Special Research Area in the National Park of Sebangau peat-swamp forest in Central Kalimantan Province, Indonesia (Fig. 1. and see Supplementary Information, 1. Research Site, Fig. 1. for further site description), which is managed by CIMTROP-UPR (Centre for International Co-operation in Sustainable Management of Tropical Peatland—University of Palangka Raya). The research camp is located in the northeastern Sebangau Forest within Sebangau National Park. The Orangutan Behaviour Project, established by Dr. Morrogh-Bernard and collaborators, operates within a defined 3 km × 3 km study area surrounding the camp and has been actively collecting data on orangutan behaviour and ecology since 2003. Throughout this time, over 100 orangutan individuals have been studied, and data from over 20,000 h of focal follows have been collected.

### Data collection

The long-term feeding ecology data used in this study were collected between 2003 and 2023 using standardised orangutan data-collection protocols^31,32^. These included the use of focal follows^33^ conducted by two-person teams. Focal follows could continue for up to ten consecutive days per focal individual. Feeding data were collected from these individuals starting from when they got up and finishing when they made their night nest, with focal follows resumed the next morning before individuals left their nest.

Behavioural data were recorded at 5-min intervals and included several behavioural aspects of orangutans’ daily activities. Feeding data were collected continuously from 2003 to 2011 and subsequently at 5-min intervals. Feeding data included species name of any resources ingested, as well as the part eaten i.e., fruit (ripe or unripe), seed, pulp, skin, pith or bark. For data analysis, all feeding data across the 20 years were consolidated. Each plant species was then further categorised, where possible, by its phenology.

### Ethnomedicinal data

To gather ethnomedicinal information on plant resources present in the Bornean orangutan diet, we conducted semi-structured interviews with two co-authors, Hendri Shagara (HS) and Iwan Shinyo (IS), whose ethnobotanical knowledge reflects that used within the local community. Both have worked as field staff and botanical guides for the Borneo Nature Foundation at the study site for over a decade. Interviews included questions on the local names of plants consumed by orangutans, medicinal parts used, associated ailments, and methods of preparation. This format also allowed for open-ended discussion, providing deeper insight into traditional uses, known medicinal properties, and local ethnobotanical knowledge. Responses were analysed thematically by coding for medicinal uses, preparation methods, and plant parts utilised. These were then organised into broader themes (e.g. application method, ailment treated) to systematically identify plants with reported therapeutic properties. Next, we integrated these data with existing ethnomedicinal records from Dayak communities near Sebangau National Park, where over 200 culturally significant plant species have been identified^34^.

### Resources of interest

From our ethnomedicinal dataset, we identified putatively medicinal resources of interest (ROIs) in the local orangutan diet by cross-referencing ethnomedicinal information with the site’s orangutan feeding database. To generate this list, we included only plants used in ethnomedicinal ingestion-based treatments and excluded those used solely for topical anointment (e.g., applied externally). This criterion was applied to ensure consistency with the ingestion-based feeding behaviours recorded in our dataset and to avoid conflating different modes of administration, which may involve distinct pharmacological mechanisms. Accordingly, ethnomedicinal references are used to provide contextual information on potential bioactivity rather than to imply direct equivalence in therapeutic function across modes of use. ROI were further restricted to cases where the plant parts used in local medicine matched those consumed by orangutans. For instance, if interview responses indicated the use of a species leaves, only observations that referenced the species’ leaves were included in our analysis.

### Botanical information and taxonomic certainty

Plant taxa were identified in the field by experienced researchers using diagnostic morphological characteristics and locally recognised vernacular names, and were cross-referenced with existing datasets from the study site. Due to the complexity of tropical flora and the need for further taxonomic verification, local names were primarily used in the analysis to ensure consistency and to minimise potential misidentification. Specifically, *Artobotrys roseus* and *Xanthophyllum ellipticum* species-level identification could not be fully confirmed at the time of analysis. These taxa were identified to the closest possible taxonomic resolution based on field identification and were retained in the analyses to represent observed medicinal and foraging behaviour, with formal herbarium verification pending.

### Data analysis

## Dataset preparation

To create a usable dataset for our analyses, we ordered feeding data by date. Then, within each day-cluster, we extracted feeding data for each focal orangutan and created separate individual feeding lists, further ordering these new datasets chronologically by food item consumed. We then extracted from this ordered data on all days when an individual consumed at least one ROI. What remained was our base dataset for both analyses. For Collocation analysis, we incorporated a step generating a list of every permutation of resource pairing for each of these remaining days, taking the order of ingestion into account (see Supplementary Information, 2. Supplementary Methods, Table 1. for more details). From this list of resource pairings, we extracted all bigram combinations that contained at least one ROI. By structuring our dataset in this way, we aimed to more effectively identify meaningful associations that could reflect medicinal combinations. For APRIORI, we kept the ordered diet lists, which contained one ROI, but did not create bigram pairings.

### Multiple distinctive collocation analysis

We applied multiple distinctive collocation analysis (MDCA) to identify statistically significant paired co-occurrences (bigrams) of food items in the orangutan feeding data, focusing on combinations involving at least one ROI. MDCA is suitable for large, non-random datasets^35^ and uses exact binomial tests to detect whether specific resource pairings occur more or less frequently than expected by chance^27,36^. Log-transformed ‘pbin’ values indicate the strength and direction of these associations, with pbin > 1.3 representing statistical significance. Full data preparation protocols and analytical procedures are provided in Supplementary Materials (2. Supplementary Methods).

### APRIORI analysis

To complement MDCA and capture more complex multi-item associations in orangutan feeding behaviour, we also applied APRIORI analysis, commonly used to detect frequent item-sets and generate association rules in transactional data^37–40^. This method identifies combinations of resources that co-occur across feeding events more frequently than expected, allowing us to explore dietary patterns beyond simple pairings and uncover recurring sets of potentially therapeutic items. Our APRIORI dataset was identical to the base dataset used to prepare bigrams for MDCA, and used the same list of ROIs, to facilitate parallel analysis^27^. The dataset was then processed through the APRIORI algorithm in R (version 4.0.5, R Core Team, 2021), with the results accessible via ‘PANacea’ (See Supplementary Information, 2. Supplementary Methods, sections “Ethnomedicinal data” and “Resources of interest”).

APRIORI is a market basket analysis, which can help uncover more complex patterns involving multiple food items, with adjustable metrics. These criteria enable users to filter the feeding data and define the analysis’s robustness, while rule length specifies the number of foods in the left-hand side (LHS) of the if–then rule. The right-hand side (RHS) of the rule will always generate a singular food item. For instance, if the rule length is three, an output might appear as: IF food items A, B, and C were consumed, THEN food item D was also likely consumed. If both confidence and lift are high for this association rule, it would strongly suggest that an individual consuming A, B, and C would also consume D. Increasing the number of items (rule length) in the LHS enhances the significance of the combination by reducing the likelihood of random opportunistic ingestion of each resource in the generated combination.

In our study, we applied a minimum threshold of support = 0.01, confidence = 0.6, and lift = 1, with a rule length of 25 to the full dataset in APRIORI analysis and again focused specifically on combinations that included at least one ROI. The Support metric represents the number of times the association appears in the data. Here, we use a Support threshold of 0.01 to capture a wide range of potential patterns and associations in the data, including both frequent and less frequent item sets. This metric helps when exploring diverse relationships without excluding potentially interesting but less common patterns. The Confidence metric of 0.6, scaled between 0 and 1, is to be interpreted as a percentage (0 = 0%, 1 = 100%). This threshold ensures that the association rules generated are statistically significant and reliable. While confidence provides an indication of the strength of associations, it is also influenced by dataset size. The Lift value of 1 adjusts for confidence and is therefore critical for interpreting datasets. To identify non-random dietary associations, we used Lift as a measure of co-occurrence strength between food items, which is particularly well suited to larger datasets with numerous observations but low frequencies of individual items or combinations. Lift quantifies the extent to which two items co-occur more frequently than expected under independence, given their baseline consumption rates. Values greater than 1 indicate positive association beyond expected co-occurrence, suggesting non-random pairing. (See Supplementary Methods, Fig. 2). By accounting for differences in individual item frequencies, this metric reduces sensitivity to common dietary components that are frequently consumed independently rather than in specific combinations.

However, spatial co-occurrence and phenological synchrony may still contribute to observed patterns, particularly where food species are co-available within shared microhabitats or overlapping periods of resource availability. Although these factors cannot be explicitly tested in the present dataset, the use of Lift as a key measure of association strength helps to reduce, though not eliminate, the influence of availability-driven co-occurrence by accounting for expected co-occurrence under conditions of independence. The generalised dietary pairing analysis provided in the Supplementary Material 4 and 5 offers additional contextual information on overall dietary structure, but does not alter the primary interpretation of SMRCH-derived associations. Accordingly, observed associations should be interpreted as deviations from statistical independence in dietary consumption rather than direct evidence of behavioural choice alone.

### Ethnomedicinal and pharmacological literature review

Finally, we conducted a post-hoc literature review to further investigate the ethnomedicinal and pharmacological properties of the plants strongly associated with ROIs. Specifically, we focused on the resources that combined with ROIs in the top five non-random resource pairings identified by MDCA and APRIORI. For each of these species, we searched for ethnomedicinal reports of traditional uses and pharmacological studies which assessed bioactivity.

To compile this information, we conducted a targeted search of peer-reviewed botanical publications. The systematic literature search was conducted following established best practices for botanical and pharmacological research^41^. Searches were performed by combining scientific plant names (genus and/or species), common names, and terms related to medicinal properties or bioactive constituents. Example search phrases included “*Fibraurea tinctoria* medicinal properties,” to increase retrieval of relevant studies, additional terms were used to specify plant parts (e.g., leaf, bark, root). Studies were included if they provided ethnopharmacological context, laboratory-based phytochemical or pharmacological analyses, and clear methodological descriptions, with preference given to recent, peer-reviewed publications from reputable journals.

#### Ethical note

Ethical approval for this study was granted by the University of Exeter (4746125). The study was purely observational and adhered to the standardised orangutan data-collection protocols^31,32^. All research complied with the American Society of Primatologists (ASP) Principles for the Ethical Treatment of Non-Human Primates (2021), and the ASP Code of Best Practices for Field Primatology (2014). All applicable international and national guidelines were followed. The two-person teams conducting focal follows consisted of trained field assistants and researchers working as part of the research team. These individuals were not study participants. Field assistants H. Shagara and I. Shinyo are included as co-authors and contributed to data collection. No human subjects were involved in this research, and no personal or sensitive data were collected. All individuals involved in data collection participated with appropriate consent in accordance with institutional guidelines. The authors report no conflict of interest.

## Results

In total, we extracted 12,236 distinct feeding events by 55 individual orangutans on 2419 days over 20 years. Overall, individuals fed on 202 distinct plant species and multiple different plant parts, including fruits, leaves, pith, roots, flowers, sap, and bark.

Our ethnomedicinal survey, informed by the extensive data compiled by Badri et al.^34^ and interviews with HS and IS, identified 64 plant species that were both cited as medicinally significant to Dayak communities and recorded in the orangutan feeding dataset. From these, we selected 19 resources as ROIs for further analysis based on their recurrent use, established medicinal properties, and relevance across both datasets. Table 1 presents the species comprising the 19 Resources of Interest (ROIs), with the full list of the 19 overlapping species parts provided in Supplementary Information 3 and Ethnobotanical Information Table 1. A subset of taxa (3 out of 9 species; or 6 of 19 ROIs) were identified only to the genus level, which constrains functional interpretation. While genus-level identification may offer limited contextual support^42–46^, pharmacological properties cannot be assumed to be consistent across all species within a genus, particularly where phytochemical data remain incomplete. In line with this, these taxa are interpreted cautiously and presented as candidate components within non-random combinations, reflecting structured co-consumption patterns rather than direct evidence of specific pharmacological activity, and are therefore treated as hypothesis-generating cases requiring species-level validation.

**Table 1:** The nine species contributing to the 19 ROIs. Distinct usable plant parts (e.g., leaf, bark, sap) were treated as separate ROIs, yielding 19 ROI species–part combinations listed in the Supplementary Information. ROIs met three criteria: (1) they were orally consumed by orangutans, (2) their ethnomedicinal use was consistently documented across interviews with H. Shagara, I. Shinyo, and the Badri et al.^34^ dataset, and (3) they were taxonomically identified to at least the genus level. These criteria ensured that selected species were both biologically relevant and reliably documented.

Taxon (vernacular)	Scientific name (Family)	Plant part(s)	Indication(s)	Ethnomedicinal preparation/Administration
Ehang	*Diospyros siamang* (Ebenaceae)	Bark	Stomach problems, diarrhea	Bark decoction taken orally
Jelutong	*Dyera* spp. (Apocynaceae)	Young Leaf	Wounds	Leaf chewed, applied as poultice
Kapurnaga Kalakei	*Calophyllum* spp. (Calophyllaceae)	Leaf, sap	Thrombocytopenia, allergies	Leaves eaten raw; sap in oil rubbed on rash
Kayu Tulang	*Baccaurea stipulata (Phyllanthaceae)*	Whole plant	Nephrolithiasis (kidney stones)	Decoction of plant (alone or with other herbs, e.g.Orthosiphon stamineus) taken orally
Liana Kalalawit Hitam	*Artabotrys roseus* (Annonaceae)	Whole plant	Cardio-hepatic disorders, hypertension	*Not recorded*
Liana Kalalawit Merah	*Uncaria* spp. (Rubiaceae)	Stem, root	Hypertension, hepatic disorders, cancer	Decoction taken orally
Liana Kuning	*Fibraurea tinctoria* (Menispermaceae)	Bark, stem, root, leaf	Malaria, typhoid, hepatitis B, jaundice	Bark decoction drunk; leaves steamed and inhaled
Mentawa	*Lepidaria* spp. (Loranthaceae)	Whole plant, floral water	Eye disease, tumours, child-birth recovery	Floral water as eyedrops; whole-plant decoction taken orally
Ponak	*Tetramerista glabra* (Tetrameristaceae)	Leaf	Dermatitis; diabetes	Leaves chewed and rubbed; leaf decoction drunk

### Multiple distinctive collocation analysis

Our MDCA analysis was run on bigrams which included at least one of the 19 ROIs identified in Table 1. The MDCA analysis produced a total output of 16,569 distinct bigrams. Of these, 324 had pbin values > 1.3 (*P* < 0.05). The pair with the highest attraction was *Mezzettia parviflora* and *Fibraurea tinctoria* (pbin = 98.73). The top 25 non-random bigrams are shown below in Table 2.

**Table 2:** The top 25 bigrams from MDCA with pbin values > 1.3.

Pairwise association rank	Resource 1	Resource 2	Pbin value
1	*Mezzettia* *parviflora*-Fruit	*Fibraurea* *tinctoria*–Leaves	98.73
2	*Gnetum* spp*.*–Fruit	*Fibraurea* *tinctoria*–Fruit	83.74
3	*Artobotrys* *roseus*–Fruit	*Artobotrys* *suaveolins*–Fruit	77.57
4	*Alyxia* spp*.*–Leaves	*Artobotrys* *roseus*–Fruit	62.42
5	*Fibraurea* *tinctoria*–Leaves	*Xylopia* *fusca*–Fruit	57.60
6	*Tetramerista* *glabra*–Leaves	*Mezzettia* *parviflora*–Fruit	57.25
7	*Pandanus* spp*.*–Pith	*Fibraurea* *tinctoria*–Leaves	51.39
8	*Fibraurea* *tinctoria*–Leaves	*Xanthophyllum* *ellipticum*–Leaves	49.33
9	*Fibraurea* *tinctoria*–Fruit	*Dyera* *lowii*–Fruit	48.04
10	*Fibraurea* *tinctoria*–Fruit	*Termites*–Invertebrates	45.72
11	*Artobotrys* *roseus*–Fruit	*Termites*–Invertebrates	44.52
12	*Elaeocarpus* *mastersii*–Fruit	*Fibraurea* *tinctoria*–Leaves	43.82
13	*Mesua* spp*.*–Fruit	*Fibraurea* *tinctoria*–Fruit	42.49
14	*Garcinia* *bancana*–Fruit	*Fibraurea* *tinctoria*–Fruit	37.82
15	*Fibraurea* *tinctoria*–Leaves	*Gnetum* spp.–Fruit	37.05
16	*Tetramerista* *glabra*–Fruit	*Fibraurea* *tinctoria*–Leaves	33.94
17	*Tetramerista* *glabra*–Leaves	*Sandoricum* *beccanarium*–Fruit	29.30
18	*Fibraurea* *tinctoria*–Leaves	*Termites*–Invertebrates	28.52
19	*Palaquium* *cochlearifolium*–Fruit	*Fibraurea* *tinctoria*–Leaves	28.32
20	*Sandoricum* *beccanarium*–Fruit	*Fibraurea* *tinctoria*–Fruit	27.26
21	*Diospyros* *bantamensis*–Leaves	*Fibraurea* *tinctoria*–Leaves	26.73
22	*Fibraurea* *tinctoria*–Leaves	*Alyxia* spp*.*–Leaves	26.16
23	*Palaquium* *cochlearifolium*–Flowers	*Fibraurea* *tinctoria*–Leaves	25.59
24	*Dyera* *lowii*–Bark	*Diospyros* *siamang*–Bark	25.49
25	*Xanthophyllum* *ellipticum*–Leaves	*Callophyllum* spp.–Leaves	25.35

### APRIORI

For the APRIORI analysis, we used the same base dataset as our MDCA analysis. The algorithm produced 7 association rules with rule lengths between 2 and 5 at our set metrics (Table 3). The rule with the highest lift (5.72) was *Alyxia* spp. + *Willu*ghbeia spp → Fibra*urea tinctoria*, with a confidence of 62.5%.

**Table 3:** Non-random medicinal resource combinations from the ROI dataset identified using APRIORI analysis (minimum support = 0.01, confidence = 0.6, lift = 1, rule length = 2–5). Within ‘Rules’, items on the left (LHS) joined by ‘+’ indicate a combination that is non-randomly associated with the item on the right (RHS) after ‘→’.

Pairwise association rank	Rules	Support	Confidence	Lift	Count
1	{*Alyxia* spp.—Leaves} + {*Willughbeia* spp.—Fruit} → {*Fibraurea tinctoria*—Leaves}	0.01	0.63	5.72	30
2	{*Xylopia fusca*—Fruit} + {*Fibraurea tinctoria*—Leaves} → {*Mezzettia parviflora*—Fruit}	0.01	0.79	2.59	37
3	{*Fibraurea tinctoria*—Leaves} + {*Pandanus* spp.—Pith} → {*Mezzettia parviflora*—Fruit}	0.01	0.69	2.29	34
4	{*Fibraurea tinctoria*—Leaves} + {*Pandanus* spp.—Pith} → {Termites—Invertebrates}	0.01	0.76	2.15	37
5	{*Xanthophyllum ellipticum*—Leaves} + {*Fibraurea tinctoria*—Leaves} → {Termites—Invertebrates}	0.01	0.73	2.08	44
6	{*Fibraurea tinctoria*—Fruit} → {Termites—Invertebrates}	0.02	0.68	1.92	63
7	{*Fibraurea tinctoria*—Leaves} + {*Tetramerista glabra*—Fruit} → {Termites—Invertebrates}	0.02	0.65	1.86	49

### Ethnomedicinal and pharmacological literature review

Results of our ethnomedicinal and pharmacological literature review can be found in Table 4. Resources selected for this review came from the top 5 ranked non-random combinations in both our MDCA and APRIORI analyses. While the ROIs were selected based on prior ethnomedicinal knowledge, many of the species most frequently co-consumed with these ROIs had not been identified in our ethnomedicinal interviews or literature review. Prioritising these resources for further review enabled an assessment of whether these commonly combined resources might have complementary or synergistic effects that complement or enhance those of the ROIs.

**Table 4:** Ethnomedicinal uses and pharmacological properties of plant species from the Top 5 ranked non-random combinations (MDCA and APRIORI).

Species classification	Ethnomedicinal uses	Known pharmacological properties
*Fibraurea tinctoria*	All parts of the plant, leaves, stems, roots, and bark, are used in traditional medicine to treat dysentery, diabetes, jaundice, and malaria^25,34,47–49^	*Fibraurea tinctoria* has diverse pharmacological properties, including analgesic, antipyretic, antidote, anti-inflammatory, and diuretic effects^25,47,49–51^. Among 38 plant species tested in South Vietnam, it showed the strongest antimalarial activity^49^. Fikriah and Sawitri^48^ further suggest its extracts may enhance artemisinin efficacy, highlighting potential synergistic interactions. Notably, Laumer et al.^7^ documented the first systematic use of a biologically active plant by great apes, observing a flanged male Sumatran orangutan applying chewed *Fibraurea tinctoria* leaves directly to a three-day-old facial wound
*Alyxia* spp.	*Alyxia* spp. have well-documented ethnomedicinal uses, particularly in Southeast Asia and Australasia. They are traditionally employed to treat fevers, stomachaches, colic, flatulence, dysentery, thrush, bronchitis, gonorrhea, rheumatism, and other inflammatory conditions^52^. Preparations include oral remedies, topical applications, leaf or bark infusions, and occasionally smoke for headaches	*Alyxia* spp. contain bioactive compounds with anti-inflammatory and antimicrobial activity^53^. Endophytic fungi associated with these plants produce additional antimicrobial compounds effective against pathogens like *Staphylococcus aureus* and *Escherichia coli*^54^ This demonstrated plant–microbe synergy makes *Alyxia* spp. a strong candidate for investigating potential non-random, synergistic interactions with other species identified in our analyses, with promising ethnomedicinal and pharmacological potential
*Xylopia* spp.	*Xylopia* spp. have been traditionally used to treat conditions such as syphilis, malaria, fungal infections, cough, stomachache, and rheumatism^55^, while *Xylopia fusca* is specifically used as an antidote for insect bites^56^	*Xylopia* spp. exhibit a wide range of biological activities, including antibacterial, antimicrobial, antifungal, antioxidant, and antitumor effects^55^. The chemical composition of *Xylopia fusca* essential oils varies by plant part, with leaf oils containing compounds which exhibit antimicrobial, anti-inflammatory, and cytotoxic effects, and twig oils containing constituents with antioxidant, antifungal, and anticancer properties^57^
*Pandanus* spp.	*Pandanus* spp. is used to treat genito-urinary diseases, primarily urinary tract infections^58^	*Pandanus* spp. extracts from leaves, roots, flowers, and fruits have shown a wide range of pharmacological activities, including antiepileptic, antioxidant, anti-inflammatory, analgesic, antidiabetic, diuretic, hepatoprotective, fertility-regulating, aphrodisiac, antimicrobial, antifungal, antiviral, anthelminthic, antitumor, and CNS depressant effects. Findings evidenced reduced blood glucose in diabetic models, strong analgesic and anti-inflammatory effects comparable to standard drugs, and antiviral activity against herpes simplex and influenza viruses^59^
*Willughbeia* spp.	*Willughbeia* spp. are used in traditional Dayak medicine in Kalimantan, Indonesia, to treat diarrhea^60^ and have also been reported for managing dementia, heartburn, cutaneous abscesses, and as a diuretic^61^. However, while some species have documented uses, further investigation is needed to explore the pharmacological potential of other *Willughbeia* species	*Willughbeia* spp. extracts displayed anti-cholinesterase activity, meaning they can block enzymes that break down the neurotransmitter acetylcholine^61^. Stem bark compounds, including lignan derivatives, have also shown moderate anticancer effects against HeLa cells, a type of human cancer cell line Arung et al.^42^
*Mezzettia parviflora*	The bark of *Mezzettia parviflora* has traditionally been used in Southeast Sulawesi, Indonesia, to treat tumors, asthma, high cholesterol, and diabetes^62^. These conditions are often linked to cellular damage caused by free radicals, which arise from normal metabolic processes as well as external factors such as pollution, UV radiation, pesticides, industrial chemicals, and tobacco smoke	Murdifin et al.^62^ found that wood bark extracts of *Mezzettia parviflora* show strong antioxidant activity, attributed to their high condensed tannin content. Endophytic fungi from *Mezzettia parviflora* also produce antioxidant compounds, reinforcing its pharmacological potential^63^. These results highlight *Mezzettia parviflora* as a valuable source of natural antioxidants and suggest that its partnership with endophytic fungi evidence its potential for synergistic applications
*Gnetum* spp.	The genus *Gnetum* has been traditionally valued both as a medicinal resource and as a food source to improve nutrition and promote health. The leaves are commonly incorporated into soups, stews, and vegetable salads^44^	Phytochemical analyses of *Gnetum* spp. reveal the presence of alkaloids with anti-inflammatory activity^64,65^
*Artobotrys roseus*	*Artobotrys* spp. extracts have traditionally been used to treat fevers, microbial infections, ulcers, liver disorders, and other ailments, with root and fruit extracts specifically applied against malaria^66^. However, further research is needed on *Artobotrys roseus* in particular	*Artobotrys* spp. contain bioactive compounds including flavonoids, alkaloids, terpenoids, and endoperoxides, with documented antimalarial, antimicrobial, and hepatoprotective activities^66,67^
*Artobotrys suaveolins*	*Artobotrys suaveolens* is traditionally used in regulating menstruation and in treating cholera^68^	*Artobotrys suaveolens* leaf extracts show strong antidiabetic activity by enhancing glucose uptake and inhibiting key enzymes^68^

## Discussion

To the best of our knowledge, this study presents the first comprehensive investigation into the ingestion of putative medicinal resources by Bornean orangutans. Our research identified 64 resources in the orangutan diet that possess local ethnomedicinal uses and/or pharmacological properties. We then employed a systematic quantitative approach to identify non-random resource combinations in orangutan feeding behaviour, using two methodological approaches recently validated in free-ranging chimpanzees^27^. By utilising MDCA and the APRIORI algorithm, we detected non-random associations between targeted resources of interest, providing evidence of potential combinatorial medicinal behaviours in wild orangutans. This approach moves beyond isolated observations by detecting structured patterns of resource use across feeding sequences and daily intake, allowing us to identify combinations that occur more frequently than expected under independent consumption. These patterns highlight consistent associations within the diet that warrant further investigation. While the analytical framework applied here provides a robust basis for identifying non-random dietary associations, it is important to note that additional, more recent methodological developments have emerged^69^. Recent work has refined collocation analysis through the introduction of MDCA-Pr, which incorporates uncertainty estimates and enables more robust comparison of signal combinations across cohorts. Although not applied in the present study, this approach represents a promising avenue for future work.

The absence of fine-scale spatial data and complete phenological records precluded the construction of a spatial null model, limiting our ability to directly evaluate the role of resource co-availability in shaping observed dietary associations. Despite these constraints, our analytical framework captures dietary structure across two complementary temporal scales. MDCA reflects short-term sequential ingestion within feeding bouts (i.e., “recipe”-level structure), whereas APRIORI analysis captures broader patterns of co-consumption across daily intake (i.e., “dietary basket” or “dosage”-level structure). Together, these approaches provide a more nuanced representation of dietary organisation than single-scale analyses, even in the absence of spatially explicit data. The detection of associations at both within-bout and daily scales suggests non-random structuring in dietary combinations; however, these patterns may still be influenced by unmeasured spatial or ecological co-occurrence factors rather than dietary choice alone.

Nonetheless, previous work on this population has characterised phenological cycles and feeding ecology, demonstrating patterns of food preference alongside seasonal variation in resource availability^31^. Although asynchronous fruiting ensures that edible resources are available throughout much of the year, this alone does not explain the repeated or preferential use of certain taxa. Of the 19 species identified within the top combinations across both APRIORI and MDCA analyses, 13 overlap with seasonally important taxa, suggesting some influence of seasonal availability. However, the presence of non-seasonally important species, together with the detection of non-random associations, indicates that availability alone does not fully account for these patterns. The generalised dietary pairing analyses (see Supplementary Materials 4 and 5) provide additional context, with 4 of the 19 ethnobotanical MDCA and APRIORI species not overlapping with the generalised dietary analyses.

Similarly, while there is partial overlap with the most frequently consumed species identified by Morrogh-Bernard^31^, 10 of the identified taxa are not among the dominant dietary components of orangutan diets, suggesting these associations are not driven solely by general feeding frequency. Only three species overlap with the highest-density taxa in the habitat, further reducing the likelihood that patterns are explained by abundance or spatial co-occurrence alone. Instead, these results are more consistent with selective incorporation of particular taxa, indicative of non-random foraging behaviour. Although these comparisons draw on earlier datasets and should be interpreted with appropriate caution, they provide important ecological context for the present findings. Accordingly, the combinations identified here are best interpreted as candidate behaviours rather than direct evidence of self-medication, and further research is required to determine their functional significance.

Resources present in the top 5 non-random MDCA bigrams by pairwise association rank (Table 2) included *Fibraurea tinctoria*, *Mezzettia parviflora, Alyxia* spp., *Xylopia* spp., *Artobotrys roseus, Artobotrys suaveolins* and *Gnetum* spp. The binary pair of *Mezzettia parviflora* and *Fibraurea tinctoria* had the highest pbin value (98.73) and represented the most significant association in our dataset. Resources present in the top five non-random APRIORI pairings by association rank (Table 3) included *Alyxia* spp., *Fibraurea tinctoria*, *Xylopia* spp., *Willughbeia* spp*, Pandanus* spp., *Mezzettia parviflora, and Xanthophyllum ellipticum*. The bigram with the highest lift produced using the APRIORI algorithm was *Alyxia* spp. + *Willughbeia* spp. → *Fibraurea tinctoria*.

### Specific resource combinations identified

Based on the combined and compared results from analyses presented, we suggest that resources *Fibraurea tinctoria*, *Alyxia* spp., *Willughbeia spp.*, *Gnetum spp.,* and *Mezzettia parviflora* be highlighted for further targeted behavioural studies and bioactivity testing. Notably, *Fibraurea tinctoria* appeared in every non-random APRIORI (Table 3) combination using our determined metric values, and 72% of the top 25 MDCA non-random pairs (Table 2). APRIORI analysis appears to align with those presented in MDCA, specifically indicating that *Fibraurea tinctoria* is consumed in more non-random combinations with several other species. In support of our SMRCH hypothesis, these results suggest that Bornean orangutans may actively seek out certain plant species in combinations and consume them in specific sequences, consistent with what would be expected of combinatorial self-medicative behaviours.

Given that these combinations appear more often than would be expected by chance, they warrant further investigation to understand their potential synergistic effects. Our results from both analyses, together with evidence that *Fibraurea tinctoria* has ethnomedicinal uses and pharmacological properties (Table 4), highlight this species as a strong candidate for further systematic testing. Interestingly, *Fibraurea tinctoria* was recently observed being used topically by a male Sumatran orangutan to treat a three-day-old facial wound^7^. While this represents a different mode of administration than the ingestion-based behaviours analysed here, it nonetheless supports the broader pharmacological relevance of this taxon and highlights its potential functional importance in orangutan self-directed health behaviours. We now encourage further bioactivity testing, which examines possible synergistic or complementary bioactive effects when *Fibraurea tinctoria* is combined with *Alyxia* spp., *Willughbeia* spp., *Mezzettia parviflora,* and/or *Gnetum* spp. Several of these species have notable pharmacological properties on their own: *Alyxia* spp. exhibits anti-inflammatory and antimicrobial activity^53^, *Willughbeia* spp. shows anti-cholinesterase effects^61^, and *Fibraurea tinctoria* contains berberine, an alkaloid with antimicrobial and anti-inflammatory properties^49^. Together, these traits suggest that combinations of these plants could provide simultaneous anti-inflammatory, antimicrobial, neuroprotective, and wound-healing effects.

It is important to note that pharmacological properties cannot be assumed to be uniform across all species within a genus. However, available genus-wide reviews and species-level studies suggest partial overlap in bioactivity within certain genera^43,45,46^, including *Gnetum*^44^ and *Willughbeia*^42^, indicating some cross-species consistency in bioactivity, although substantial interspecific variation remains and both taxa are comparatively under-studied. Accordingly, we present genus-level taxa as candidate components within non-random combinations rather than confirmed ethnomedicinal or pharmacologically characterised resources, as their inclusion reflects structured co-consumption patterns identified by the analytical framework rather than direct evidence of medicinal use, and they are therefore treated as hypothesis-generating candidates requiring species-level validation for robust functional inference.

The identification of non-random food combinations, particularly those involving medicinal plants, supports both the SMRCH hypothesis of intentional self-medication in orangutans and the self-medicative resource combination hypothesis. However, while the analyses identify statistically robust and non-random dietary associations, these patterns do not provide direct evidence of therapeutic intent or self-medication. Instead, they indicate structured patterns of co-consumption that are consistent with, but not diagnostic of, candidate medicinal behaviour, and may also be influenced by ecological factors such as resource availability and spatial or temporal co-occurrence.

Stronger inference of therapeutic self-medication traditionally requires concurrent data on individual health status, observable symptoms, and subsequent physiological or behavioural changes following ingestion; data which is rarely available in wild populations and typically depends on long-term focal monitoring. In this context, the application of the SMRCH provides a complementary approach by enabling the identification of non-random dietary associations from existing long-term datasets, without relying on these rarely observed health-linked events.

Accordingly, our findings should be interpreted as hypothesis-generating, identifying candidate taxa and behavioural patterns that can help prioritise future targeted studies. These should ideally integrate chemical analyses with detailed behavioural and health data to validate therapeutic function, and are now being applied in this way, while also offering potential insights into novel medicinal resources and reinforcing the importance of Indigenous ecological and ethnomedicinal knowledge for biodiversity conservation and global health research.

## Supplementary Information

Below is the link to the electronic supplementary material.

Supplementary Material 1

## Data Availability

The datasets generated and analysed during this study are held by the Borneo Nature Foundation, with the principal data custodian being H. Morrogh-Bernard. Due to the conservation sensitivity of orangutan habitats and detailed feeding ecology data, the raw datasets are not publicly available but may be accessed upon reasonable request to the corresponding author. All underlying data will be made available to the editors and reviewers during peer review if requested, in accordance with ethical and conservation guidelines.

## Abbreviations

MDCA: Multiple distinctive collocation analysis
ROI: Resource of interest
SMRCH: Self-medicative resource combinational hypothesis
