# Supplementary material for: Investigating medicinal resource combinations in the Bornean orangutan diet
Source: Sci Rep. 2026 May 13;16:18690. doi: 10.1038/s41598-026-52614-4 (PMC13272804; doi:10.1038/s41598-026-52614-4)
Supplement: Supplementary file 1 — Supplementary Material 1 [file 41598_2026_52614_MOESM1_ESM.docx]

**SUPPLEMENTARY INFORMATION - Investigating Medicinal Resource Combinations in the Bornean Orangutan Diet**

**1. Research Site**


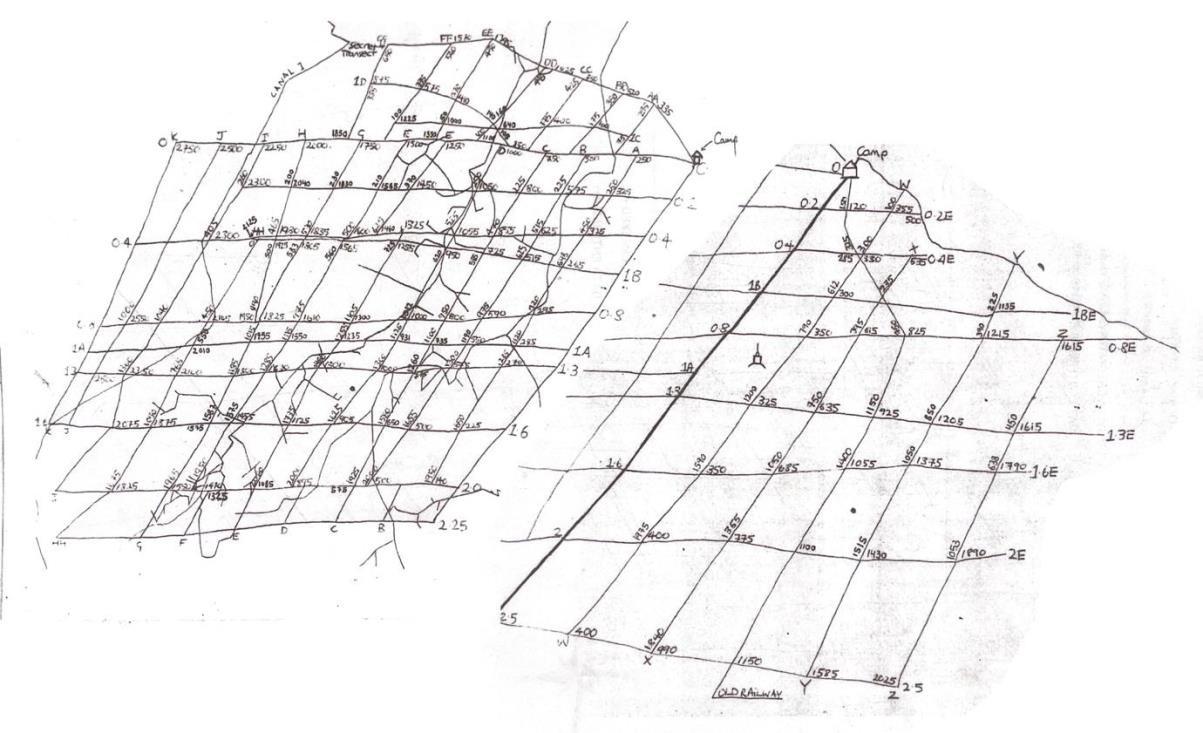


Figure 1. The Sebangau Forest Research Site, Central Kalimantan, Indonesia. West and East facing transects of research site.

**2. Supplementary Methods:**

**2.1** Following methods of Freymann et al., (2024), all feeding data in our dataset was ordered by date. Within each daily cluster, feeding data from each individual orangutan actor observed feeding during that period were extracted and compiled into individual feeding lists. Each individual’s daily feeding data were then chronologically grouped. The data was cleaned and instances where individuals consumed only one food item in a day were excluded, as these did not allow for the possibility of food combinations. When an individual consumed the same resource consecutively, the second event was removed to prevent double counting, unless it was interrupted by the consumption of a different food. If a resource was eaten twice with different foods consumed in between, it was retained in the dataset (e.g., orangutan eats food A, then food B, then food A again). From this, every permutation of food pairing for each individual was calculated, preserving the order of ingestion. For example, if orangutan X was seen eating 3 foods in the following order - Jangkang Kuning, Liana Kuning, then Pisang Pisang Besar Fruit - on day Y, the dataset for that individual-day-cluster would look like Table 1. To identify foods with known medicinal properties, we collected and consulted existing ethnobotanical and bioactivity literature (Badri et al., 2022). Putative medicinal resource here is defined as a resource, or more specifically resource part, consumed by the local population for medicinal purposes. (See supplementary information 3.1)

TABLE 1: Example of possible food permutations from a single individual on a single day

| Food 1 | Food 2 |
| --- | --- |
| Jangkang Kuning | Liana Kuning |
| Jangkang Kuning | Pisang Pisang Besar Fruit |
| Liana Kuning | Pisang Pisang Besar Fruit |


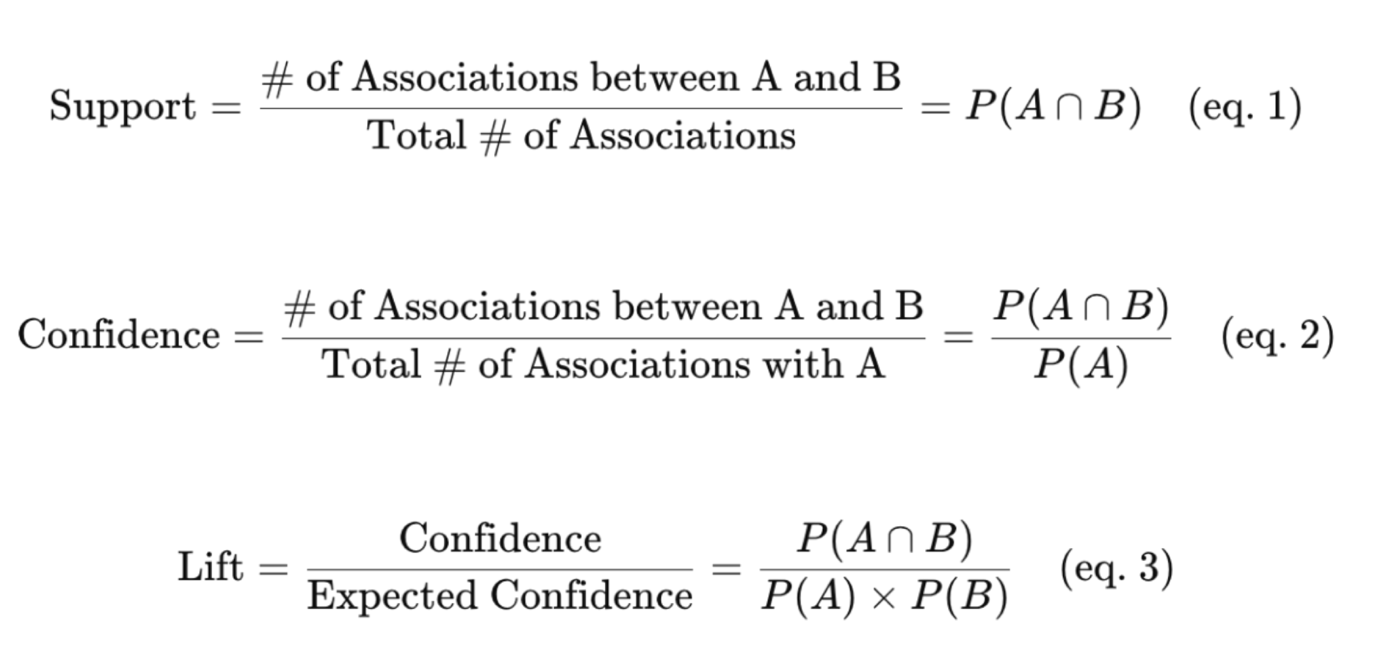


Figure 2. APRIORI metric equations

**2.2 S Gries Collocation R script**

<https://www.stgries.info/teaching/groningen/index.html>

#Gries, Stefan Th. 2014. Coll.analysis 3.5. A script for R to compute perform collostructional analyses.

#Gries Code

source("http://www.linguistics.ucsb.edu/faculty/stgries/teaching/groningen/coll.analysis.r")

#Bosshard's Code (If Gries Doesn't Work)

source("https://gitlab.uzh.ch/alexandra.bosshard/call-ocation//raw/master/coll.analysis3.2_calls.txt")

# 2 multiple distinctive collexeme analysis

# 2 3+ categories ->approximation to the multinomial test: the one-tailed exact binomial test. # 5 dec. should be enough

# choose long_bigram.txt

# order 1 alphabetically

# save into prepared txt output file

### Collocation analysis Maël###

## multiple distinctive Collocation

#Gries, Stefan Th. 2014. Coll.analysis 3.5. A script for R to compute perform collostructional analyses.

source("http://www.linguistics.ucsb.edu/faculty/stgries/teaching/groningen/coll.analysis.r")

#2 multiple distinctive collexeme analysis

#2 3+ categories ->approximation to the multinomial test: the one-tailed exact binomial test. # 5 dec. should be enough

#choose long_bigram_short.txt

# order 1 alphabetically

#save into prepared txt output file

#Bosshard's Code

#> # PRESS <Enter> #

#> # PRESS <Enter> #

#> 2## (2 chooses MDCA)

#>2## (2 chooses 3+ categories → one-tailed exact binomial test)

#>3## (Choosing 3 decimals)

# PRESS <Enter> and CHOOSE FILE ## (here “input_file_name_of_bigram_list.txt”) #

#> 1## (1 orders output alphabetically)

# PRESS <Enter> and CHOOSE FILE ## (here: “outcome_file_name_for_MDCA.txt”) #

**2.3 PANacea link to view APRIORI results and modify metrics**

Results described are available on <https://osteomics.com/PANacea_orangutan/>with the default settings. PANacea leverages the visualisation techniques for association rules available in the arulesViz package and is hosted on the Osteomics platform. (See supplementary information 2.2). PANacea’s interactive interface allows users to customise metrics through an interactive side panel and a main panel. On the “Data Exploration” tab, users can adjust Minimum Support, Minimum Confidence, Minimum Lift, and Rule Length (1–10) via the side panel.

**2.4**
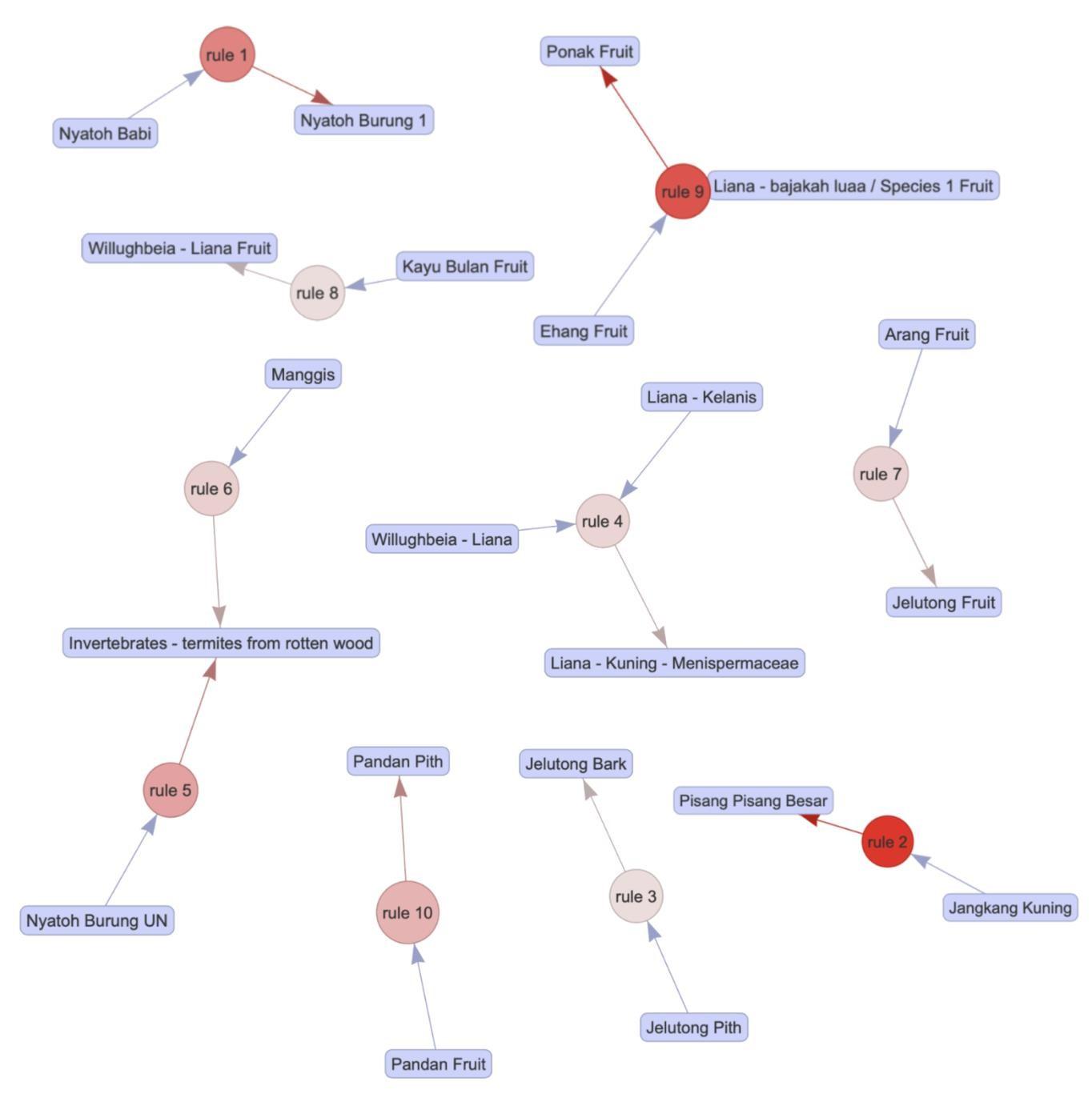


Figure 3. Example of a rule node network result on PANacea. Showing Top 10 APRIORI V1 results (ordered by lift) with minimum metrics of support = 0.01, confidence = 0.6, and lift = 1, rule length = 2-5. Rule circles are marked from a light pink to red based on confidence level, red being the highest value. Blue arrows pointing toward the rule circles indicate resources on the LHS of each equation, while red arrows pointing away from the red circle indicate the resource on the RHS.

**3. Ethnobotanical Information**

**3.1** Table of Identified Medicinal Species.

This compilation utilises the original orangutan feeding dataset spanning over 20 years. The table below compares and highlights this dataset with interview insights from Hendri Shagara and Iwan Shinyo, along with ethnobotanical data collected by Badri et al. (2022) through interviews with local ethnobotanical experts. The ROIs include: Ehang Bark, Jelutong Leaves, Kapurnaga kalakei Leaves, Kayu Tulang, Liana Kalalawit, Liana Kalalawit Hitam, Liana Kalalawit Hitam Fruit, Liana Kalalawit Merah, Liana Kalalawit Merah Fruit, Liana Kalalawit Merah Leaves, Liana Kuning Menispermaceae, Liana Kuning Menispermaceae Flowers, Liana Kuning Menispermaceae Fruit, Liana Kuning Menispermaceae Leaves, Liana Kuning Menispermaceae Pith, Liana Kuning Menispermaceae Unknown, Mentawa Flowers, Mentawa Fruit, Ponak Leaves.

Table 2. Table of Identified Medicinal Species

| **Nº** | **Taxon (vernacular)** | **Scientific name (Family)** | **Plant part(s)** | **Indication(s)** | **Preparation / Administration** | **Ethnobotanical notes** |
| --- | --- | --- | --- | --- | --- | --- |
| 1 | \| Aci \| \| --- \| | \| Garcinia sp. 1 (Clusiaceae) \| \| --- \| | Sap | Allergic‐skin eruptions | Sap mixed with cooking oil; applied topically |  |
| 2 | \| Anggrek Bawang \| \| --- \| | \| Eria sp. (Orchidaceae) \| \| --- \| | Stem | Open wounds | Stem rubbed directly on lesion |  |
| 3 | \| Anggrek Tanduk Rusa \| \| --- \| | \| (Orchidacea) \| \| --- \| | Stem, root | Dyspepsia, bloody diarrhoea | Decoction taken orally |  |
| 4 | \| Bintan Rambut Merah \| \| --- \| | \| Pandanus spp. (Pandanaceae) \| \| --- \| | Bark | Gastric disorders | Bark decoction taken orally |  |
| 5 | \| Balawan Punai \| \| --- \| | \| Tristaniopsis sp. 4 (Myrtaceae) \| \| --- \| | Bark | Gastric disorders | Bark decoction taken orally |  |
| 6 | \| Blawan Putih (fruit) \| \| --- \| | \| Tristaniopsis sp. 3 cf. merguensis (Myrtaceae) \| \| --- \| | Bark | Gastric disorders | Bark decoction taken orally |  |
| 7 | \| Ehang \| \| --- \| | \| Diospyros siamang (Ebenaceae)* \| \| --- \| | Bark | Stomach problems, diarrhea | Bark decoction taken orally | Consumed orally; included in analysis |
| 8-16 | \| “Epiphyte” complex \| \| --- \| | \| Rubiaceae sp. \| \| --- \| | Floral water / whole plant | Internal tumours | A) Floral exudate used as eyedrops B) Whole plant decocted and drunk |  |
| 17 | \| Ficus sp. 1 \| \| --- \| | \| Ficus sp. 1 (“Fig species 1”; Moraceae) \| \| --- \| | Root | Post-partum tonic | Root decoction taken by women for 40 days after delivery |  |
| 18 | \| Fungi Peat moss \| \| --- \| | \| Sphagnum spp. (Sphagnaceae) \| \| --- \| | Whole moss | Antiseptic | Moss crushed (fresh or dried) and packed on wound |  |
| 19 | \| Kulat mata palanduk / jamur lender, Fungi Peat moss \| \| --- \| | \| Calostoma insigne (Calostomataceae) \| \| --- \| | Fruiting body | Antipyretic | Consumed raw with gelatinous outer layer |  |
| 20 | \| Gandis \| \| --- \| | \| Garcinia cf. parvifolia (Clusiaceae) \| \| --- \| | Sap | Allergies | Sap rubbed on skin |  |
| 21 | \| Gantalan / Manggis September fruit \| \| --- \| | \| Garcinia sp. 3 (Clusiaceae) \| \| --- \| | Sap | Allergies | Sap rubbed on skin |  |
| 22 | \| Gulung Haduk \| \| --- \| | \| Diospyros cf. evena (Ebenaceae) \| \| --- \| | Bark | Diarrhoea | Bark decoction taken orally |  |
| 23 | \| Forest honey \| \| --- \| | \|  \| \| --- \|   - | Honey | Cough, common cold | Honey ingested neat |  |
| 24 | \| Jambu Burung 0/2 \| \| --- \| | \| Syzygium garcinifolia (Myrtaceae) \| \| --- \| | Bark | Gastric disorders | Bark decoction taken orally |  |
| 25 | \| Jambu Jambu \| \| --- \| | \| Syzygium sp. (Myrtaceae) \| \| --- \| | Bark | Gastric disorders | Bark decoction taken orally |  |
| 26 | \| Jambu Jambu 4 \| \| --- \| | \| Syzygium sp. (Myrtaceae) \| \| --- \| | Bark | Gastric disorders | Bark decoction taken orally |  |
| 27 | \| Jelutong \| \| --- \| | \| Dyera spp. (Apocynaceae) \| \| --- \| | Sap | Wounds | Sap rubbed directly |  |
| 28 | \| Jelutong \| \| --- \| | \| Dyera spp. (Apocynaceae)* \| \| --- \| | Young Leaf | Wounds | Leaf chewed, applied as poultice | Consumed orally; included in analysis |
| 29 | \| Jinjit / Bintangor \| \| --- \| | \| Calophyllum hosei (Calophyllaceae) \| \| --- \| | Sap | HIV/AIDS (folk use) | Preparation unknown |  |
| 30 | \| Kala Pimping Napu \| \| --- \| | \| Dialium patens (Fabaceae) \| \| --- \| | Root | Gastric disorders | Root decoction taken orally |  |
| 31 | \| Kalanduyung Himba \| \| --- \| | \| Ardisia cf. sanguinolenta (Primulaceae) \| \| --- \| | Bark, leaf | Diabetes, hyper-cholesterol, topical ulcers | Bark/leaf decoction drunk; pulp rubbed on lesions |  |
| 32 | \| Kamba Sulan \| \| --- \| | \| Ardisia sp. 2 (Primulaceae) \| \| --- \| | Leaf | Skin cleansing | Leaves rubbed on face |  |
| 33 | \| Kapurnaga \| \| --- \| | \| Calophyllum sclerophyllum (Calophyllaceae) \| \| --- \| | Sap | Allergies | Sap rubbed on rash |  |
| 34 | \| Kapurnaga Kalakei \| \| --- \| | \| Calophyllum sp. (Calophyllaceae)* \| \| --- \| | Leaf (sap) | Thrombocytopenia, allergies | Leaves eaten raw; sap in oil rubbed on rash | Consumed orally; included in analysis |
| 35 | \| Kayu Lalas Duan Besar \| \| --- \| | \| Syzygium cf. valevenosum (Myrtaceae) \| \| --- \| | Bark | Gastric disorders | Bark decoction taken orally |  |
| 36 | \| Kayu Lalas Daun Kecil / Helen Helen fruit \| \| --- \| | \| Syzygium sp. (Myrtaceae) \| \| --- \| | Bark | Gastric disorders | Bark decoction taken orally |  |
| 37 | \| Kayu Lalas sp \| \| --- \| | \| Syzygium sp. (Myrtaceae) \| \| --- \| | Bark | Gastric disorders | Bark decoction taken orally |  |
| 38 | \| Kayu Tulang \| \| --- \| | \| Baccaurea stipulata (Phyllanthaceae)* \| \| --- \| | Whole plant | Nephrolithiasis (kidney stones) | Decoction of plant (alone or with other herbs, e.g.Orthosiphon stamineus) taken orally | Consumed orally; included in analysis |
| 39 | \| Liana Kalalawit Hitam \| \| --- \| | \| Artabotrys cf. roseus (Annonaceae)* \| \| --- \| | Not specified | Cardio-hepatic disorders, hypertension | Not recorded | Consumed orally; included in analysis |
| 40 | \| Liana Kalalawit Merah \| \| --- \| | \| Uncaria sp. 1 (Rubiaceae)* \| \| --- \| | Stem, root | Hypertension, hepatic disorders, cancer | Decoction taken orally | Consumed orally; included in analysis |
| 41 | \| Liana Karinat \| \| --- \| | \| Ziziphus angustifolius (Rhamnaceae) \| \| --- \| | Leaf shoot | Cataract | Leaf macerate used as eyedrops |  |
| 42 | \| Liana Kelanis \| \| --- \| | \| Alyxia sp. 1 (Apocynaceae) \| \| --- \| | Bark | Anti-wrinkle face wash | Bark rubbed on skin |  |
| 43 | \| Liana Kuning \| \| --- \| | \| Fibraurea tinctoria (Menispermaceae)* \| \| --- \| | Bark, stem, root, leaf | Malaria, typhoid, hepatitis B, jaundice | Bark decoction drunk; leaves steamed and inhaled | Consumed orally; included in analysis |
| 44 | \| Loting \| \| --- \| | \| Sterculia rhoiidifolia (Malvaceae) \| \| --- \| | Sap | Gastric ulcer | Sap diluted with hot water and drunk |  |
| 45-48 | \| Lunuk group \| \| --- \| | \| Ficus spp. (Moraceae) \| \| --- \| | Root | Post-partum tonic | Root decoction drunk for 40 days; tradition attributed to observing orangutan behaviour |  |
| 49 | \| Mahadingan \| \| --- \| | \| Calophyllum sp. 2 (Calophyllaceae) \| \| --- \| | Sap | HIV/AIDS, allergy | Sap rubbed on skin |  |
| 50-52 | \| Manggis complex \| \| --- \| | \| Garcinia spp. (Clusiaceae) \| \| --- \| | Sap | Allergies | Sap rubbed on skin |  |
| 53 | \| Mangkinang \| \| --- \| | \| Elaeocarpus mastersii (Elaeocarpaceae) \| \| --- \| | Young leaf | Allergies | Leaf rubbed on skin |  |
| 54 | \| Mentawa \| \| --- \| | \| Lepidaria sp. 1 (Loranthaceae)* \| \| --- \| | Whole plant, floral water | Eye disease, tumours, child-birth recovery | A) Floral water as eyedrops B) Whole-plant decoction | Consumed orally; included in analysis |
| 55 | \| Milas \| \| --- \| | \| Syzygium sp. 14 (Myrtaceae) \| \| --- \| | Bark | Gastric disorders | Bark decoction taken orally |  |
| 56 | \| Moss (general) \| \| --- \| | \| Sphagnum spp. \| \| --- \| | Whole moss | Antiseptic | Moss crushed and packed on wound |  |
| 57 | \| Panamar Pari \| \| --- \| | \| Ampelocissus rubiginosa (Vitaceae) \| \| --- \| | Stem | Acute diarrhea | Stem chewed/eaten raw |  |
| 58 | \| Pisang Pisang Besar \| \| --- \| | \| Mezzettia parviflora (Annonaceae) \| \| --- \| | Young leaf, root corm | Antiseptic for cuts; anti-bruise poultice | Leaf applied topically; fruit, stem and blossom edible |  |
| 59 | \| Ponak \| \| --- \| | \| Tetramerista glabra (Tetrameristaceae)* \| \| --- \| | Leaf | Dermatitis; diabetes | Leaves chewed and rubbed; leaf decoction drunk | Consumed orally; included in analysis |
| 60 | \| Rotan / Uey Liling \| \| --- \| | \|  \| \| --- \|   Calamus sp.1 (Arecaceae) | Stem | Epistaxis, colds, hyperthermia | Dried stem burnt; smoke inhaled |  |
| 61 | \| Tabati Himba \| \| --- \| | \| Memecylon sp. 3 (Melastomataceae) \| \| --- \| | Young leaf | Pruritic dermatitis | Leaves pounded (often with raw rice) to powder; applied on skin |  |
| 62 | \| Tampohot Himba \| \| --- \| | \| Syzygium sp. 13 (Myrtaceae) \| \| --- \| | Bark | Gastric disorders | Bark decoction taken orally |  |
| 63 | \| Tatumbu \| \| --- \| | \| Syzygium havilandii (Myrtaceae) \| \| --- \| | Bark | Gastric disorders | Bark decoction taken orally |  |
| 64 | \| Willughbeia sp. \| \| --- \| | \| Willughbeia sp. (Apocynaceae) \| \| --- \| | Bark, root, vine sap | Diarrhea, Emergency drinking water, tonic | Bark decoction taken orally. Vine cut and water drunk; bark for ropes |  |

**4. Generalised Dietary MDCA and APRIORI Analysis Results**

In this study, we originally employed two iterations to investigate both the initial recognition of dietary pairings (Version 1/V1) and an examination involving at least one potential self-medicative source (Version 2/V2, as shown in the original manuscript) derived from ethnobotanical knowledge (Badri et al., 2022). To provide context for the plant species identified through the SMRCH analysis, we originally examined their occurrence relative to the general dietary repertoire. The generalised dietary dataset reflects overall feeding frequencies without constraints. In contrast, the SMRCH analysis identifies non-random co-ingestion patterns within a subset of feeding records that include candidate medicinal species (ROIs). The most frequently consumed plant species in the general dietary dataset differed from those identified within the SMRCH-derived combinations. This suggests that the detected associations are not solely driven by the most dominant components of the diet. However, a more rigorous comparison, for example through modelling relative frequency distributions or controlling for availability, represents an important direction for future research.

**4.1** Version 1 Results: Multiple Distinctive Collocation Analysis (MDCA) V1

Collocation MDCA V1 produced a total output of 168,940 distinct bigrams. Of these, 3,643 had pbins > 1.3 (P < 0.05). Outputs were ordered by pbin value, here we present the 25 bigrams with the highest pbin values (Table 2; range = 118.72–297.36). The pair with the highest attraction (297.36) was Mesua spp. (Tabaras akar tinggi Fruit) and Mezzettia umbellate (Pisang Pisang Kecil Fruit.)

TABLE 3: Top 25 Results of MDCA Version 1 analysis (25 most significant resource bigrams from complete data set (pbins > 1.3).)

| Resource 1 | Resource 2 | pbin value |
| --- | --- | --- |
| Mesua sp. 1 Fruit | Mezzettia umbellata Fruit | 297.361777025 |
| Invertebrates - termites from rotten wood | Diospyros siamang Fruit | 244.201332885552 |
| Lithocarpus cf. dasystachys Fruit | Garcinia bancana Fruit | 241.261903584442 |
| Willughbeia sp. 1 Liana Fruit | Mesua sp. 1 Fruit | 227.980076055951 |
| Invertebrates - termites from rotten wood | Invertebrates - ants | 227.19579615 |
| Pandanus sp. Pith | Pandanus sp. Fruit | 209.178604067 |
| Willughbeia sp. 1 Liana Fruit | Mezzettia leptopoda parviflora Fruit | 200.314103995391 |
| Palaquium cochlearifolium Fruit | Gnetum sp. 1 Fruit | 191.23819663969 |
| Mezzettia leptopoda parviflora Fruit | Pandanus sp. Pith | 189.930085135082 |
| Diospyros bantamensis Fruit | Ctenolophon parvifolius Fruit | 167.667842962 |
| Mezzettia leptopoda parviflora Fruit | Sandoricum beccanarium Fruit | 167.008804681477 |
| Gnetum sp. 2 Fruit | Fibraurea tinctoria Fruit | 153.496806357102 |
| Syzygium garcinifolia Fruit | Polyalthia hypoleuca Fruit | 147.110642745144 |
| Sandoricum beccanarium Fruit | Palaquium cf. xanthochymum Fruit | 145.235756123 |
| Palaquium ridleyii xanthochymum Fruit | Garcinia bancana Fruit | 143.088454921178 |
| Dyera lowii Pith | Dyera lowii Bark | 134.50105592 |
| Palaquium ridleyii xanthochymum Fruit | Diospyros bantamensis Fruit | 131.814715863854 |
| Willughbeia sp. 1 Liana Fruit | Water Water | 130.7591 |
| Gnetum sp. 1 Fruit | Xylopia fusca Fruit | 128.29037553208 |
| Willughbeia sp. 1 Liana Fruit | Campnosperma coriaceum Fruit | 128.188756256014 |
| Zizyphus angustifolius Fruit | Dyera lowii Fruit | 127.98023091375 |
| Mezzettia leptopoda parviflora Fruit | Palaquium cochlearifolium Flowers | 127.505597099927 |
| Palaquium ridleyii Fruit | Palaquium cf. xanthochymum Fruit | 119.33807536 |
| Invertebrates - ants | Diospyros siamang Fruit | 119.110094069135 |
| Epiphyte Leaves | Diospyros siamang Fruit | 118.718201820651 |

**4.2** Version 1 Results: APRIORI Analysis V1

We assigned a minimum threshold of support = 0.01, confidence = 0.6, and lift = 1, rule length = 2-5 and extracted the top 25 generated rules out of 1,136 combinations, presented in Table 4. The rule with the highest lift (19.48) was Palaquium pseudorostratum (Nyatoh Babi) → Palaquium cf. xanthochymum (Nyatoh Burung), which has a 72.5% confidence outcome. This implies that throughout the data collection period of 20 years, when Nyatoh Babi was consumed, it was exceedingly likely that Nyatoh Burung was then eaten by that individual. Such a high lift value illustrates the strength of the association beyond random chance .

TABLE 4: Top 25 Results of APRIORI Version 1 analysis (ordered by lift, support = 0.01, confidence = 0.6, and lift = 1, rule length = 2-5)

| Rules | Support | Confidence | Lift | Count |
| --- | --- | --- | --- | --- |
| {Palaquium pseudorostratum} => {Palaquium cf. xanthochymum} | 0.0100869565217391 | 0.725 | 19.4801401869159 | 29 |
| {Xylopia fusca} => {Mezzettia leptopoda parviflora} | 0.0111304347826087 | 0.8 | 9.16334661354582 | 32 |
| {Dyera lowii Pith} => {Dyera lowii Bark} | 0.0121739130434783 | 0.614035087719298 | 7.84600389863548 | 35 |
| {Alyxia sp. 1 - Willughbeia sp. 1 Liana} => {Fibraurea tinctoria} | 0.0104347826086957 | 0.625 | 5.72253184713376 | 30 |
| {Palaquium ridleyii xanthochymum} => {Invertebrates - termites from rotten wood} | 0.0208695652173913 | 0.697674418604651 | 5.61852648035959 | 60 |
| {Garcinia bancana} => {Invertebrates - termites from rotten wood} | 0.0191304347826087 | 0.632183908045977 | 5.09111690653273 | 55 |
| {Diospyros confertiflora Fruit} => {Dyera lowii Fruit} | 0.0118260869565217 | 0.62962962962963 | 4.3514066951567 | 34 |
| {Polyalthia glauca Fruit} => {Willughbeia sp. 1 - Liana Fruit} | 0.0121739130434783 | 0.614035087719298 | 4.19323248739426 | 35 |
| {Diospyros siamang Fruit, Gnetum sp. 1 Fruit} => {Tetramerista glabra Fruit} | 0.0114782608695652 | 0.767441860465116 | 4.18670844181634 | 33 |
| {Pandanus sp. Pith} => {Pandanus sp. Pith} | 0.0191304347826087 | 0.670731707317073 | 3.94346351438975 | 55 |
| {Ctenolophon parvifolius Fruit} => {Diospyros bantamensis Fruit} | 0.0177391304347826 | 0.671052631578947 | 3.79032675007755 | 51 |
| {Polyalthia hypoleuca Fruit, Willughbeia sp. 1 - Liana Fruit} => {Diospyros bantamensis Fruit} | 0.015304347826087 | 0.628571428571429 | 3.55037889419029 | 44 |
| {Xylopia fusca Fruit, Parartocarpus venenosus Flowers} => {Mezzettia leptopoda parviflora Fruit} | 0.0128695652173913 | 0.925 | 3.04624856815578 | 37 |
| {Xylopia fusca Fruit, Alyxia sp. 1 Leaves} => {Mezzettia leptopoda parviflora Fruit} | 0.0114782608695652 | 0.846153846153846 | 2.78658912679531 | 33 |
| {Xylopia fusca Fruit, Dyera lowii Fruit, Willughbeia sp. 1 - Liana Fruit} => {Mezzettia leptopoda parviflora Fruit} | 0.0104347826086957 | 0.810810810810811 | 2.67019596916504 | 30 |
| {Xylopia fusca Fruit, Fibraurea tinctoria Leaves} => {Mezzettia leptopoda parviflora Fruit} | 0.0128695652173913 | 0.787234042553192 | 2.59255197289854 | 37 |
| {Parartocarpus venenosus Flowers} => {Mezzettia leptopoda parviflora Fruit} | 0.0194782608695652 | 0.756756756756757 | 2.49218290455404 | 56 |
| {Xylopia fusca Fruit, Pandanus sp. Pith} => {Mezzettia leptopoda parviflora Fruit} | 0.024 | 0.734042553191489 | 2.41737954229729 | 69 |
| {Xylopia fusca Fruit, Dyera lowii Fruit} => {Mezzettia leptopoda parviflora Fruit} | 0.0208695652173913 | 0.731707317073171 | 2.40968904534406 | 60 |
| {Xylopia fusca Fruit, Diospyros bantamensis Leaves} => {Mezzettia leptopoda parviflora Fruit} | 0.0111304347826087 | 0.727272727272727 | 2.39508486931167 | 32 |
| {Xylopia fusca Fruit} => {Mezzettia leptopoda parviflora Fruit} | 0.0820869565217391 | 0.717325227963526 | 2.36232534982261 | 236 |
| {Xylopia fusca Fruit, Tetramerista glabra Fruit} => {Mezzettia leptopoda parviflora Fruit} | 0.0236521739130435 | 0.708333333333333 | 2.33271286750668 | 68 |
| {Garcinia bancana Fruit, Pandanus sp. Pith} => {Invertebrates - termites from rotten wood} | 0.0111304347826087 | 0.82051282051282 | 2.33100233100233 | 32 |
| {Pandanus sp. Pith, Mezzettia leptopoda parviflora Fruit, Willughbeia sp. 1 - Liana Fruit} => {Invertebrates - termites from rotten wood} | 0.0139130434782609 | 0.816326530612245 | 2.31910946196661 | 40 |
| {Pandanus sp. Pith, Water} => {Invertebrates - termites from rotten wood} | 0.0194782608695652 | 0.811594202898551 | 2.30566534914361 | 56 |

**5. Discussion of V1 Results**

**5.1** Collocation MDCA Version 1 Analysis

Collocation MDCA V1 explored all food combinations resulting in Mesua spp. (Tabaras akar tinggi Fruit) and Mezzettia umbellate (Pisang Pisang Kecil Fruit) (see Table 2) as the highest non-random binary pairing, suggesting non-random patterns in their foraging behaviour. Following the identification of the top 5 non-random pairings produced by MDCA V1 (Table 2), subsequent research revealed that resources *Garcinia bancana* (manggis fruit) and *Willughbeia* spp. (*Willughbeia Liana*) possess medicinal properties. Manggis Fruit (*Garcinia bancana*) contains anti-plasmodial and antioxidant activity (Rifaldi et al., 2023). *Willughbeia Liana* (*Willughbeia* spp.) is used to treat dementia, heartburn, and cutaneous abscesses, as well as being used as a diuretic. Compounds extracted demonstrated inhibitory activities against acetylcholinesterase (AChE) and butyrylcholinesterase (BChE) in vitro for Anti-cholinesterase activity (Can et al., 2018). It is important to note that while uses are mentioned for some *Willughbeia* species there is a need for further exploration of other species.

**5.2** APRIORI Version 1 Analysis

In APRIORI V1 analysis, the highest lift combination showed *Palaquium pseudorostratum* (Nyatoh Babi) → *Palaquium cf. xanthochymum* (Nyatoh Burung) with a 72.5% confidence outcome rendering them noteworthy species for further investigation. Following the identification of the top 5 non-random pairings produced, subsequent research revealed resources *Xylopia* spp. (Jangkang Kuning), *Alyxia* spp. (Liana Kelanis), *Willughbeia* spp. (*Willughbeia Liana*), and *Fibraurea tinctoria* possess medicinal properties (see Table 4 in original paper).
